# A Lethal Case of Disseminated *Cladosporium allicinum* Infection in a Captive African Bullfrog

**DOI:** 10.3390/jof9020191

**Published:** 2023-02-01

**Authors:** Andrea Grassi, Matteo Gambini, Marianna Pantoli, Simona Toscano, Anna Albertetti, Deborah Maria Del Frassino, Iniobong Chukwuebuka Ikenna Ugochukwu, Orazio Romeo, Domenico Otranto, Claudia Cafarchia

**Affiliations:** 1 I-Vet Diagnostica Veterinaria, 25020 Brescia, Italy; 2 Istituto Zooprofilattico Sperimentale della Lombardia e dell’Emilia Romagna, 27100 Pavia, Italy; 3 Ambulatorio Veterinario Toscano, 10154 Torino, Italy; 4 Dipartimento di Medicina Veterinaria, Università degli Studi di Bari Aldo Moro, 70124 Bari, Italy; 5 Department of Veterinary Pathology and Microbiology, University of Nigeria, Nsukka 400001, Nigeria; 6 Department of Chemical, Biological, Pharmaceutical and Environmental Sciences, University of Messina, 98166 Messina, Italy; 7 Faculty of Veterinary Sciences, Bu-Ali Sina University, Hamedan 6517833131, Iran

**Keywords:** chromoblastomycosis, phaeohyphomycosis, *Cladosporium allicinum*, African bullfrog

## Abstract

*Cladosporium* infections have a poor prognosis in animals, most likely due to a lack of knowledge about diagnosis and treatment. In this study, we described a case of a lethal *Cladosporium allicinum* infection in a captive bullfrog (*Pyxicephalus adspersus*) in Europe. One adult male bullfrog was referred with clinical signs of lethargy and a cutaneous nodule. Fungal infection was suspected on cytology and confirmed by histology and cultural isolation. The mold was identified by molecular methods using partial sequencing of the TEF1α gene and the ITS region of rDNA. Climbazole antifungal treatment was started but the frog died after 30 days, and necropsy was done. Pigmented hyphae and structures consistent with muriform bodies were found on a background of diffuse granulomatous inflammation at cytological and histopathological examinations. Fungal culture revealed the presence of pigmented fungi identified as *Cladosporium allicinum* only by partial sequencing of the TEF1α gene. A focally extensive granuloma with intralesional hyphae and muriform bodies effacing the architecture of head, liver, kidneys, lungs, and large intestine were retrieved after necropsy. This study is the first Italian report of the occurrence of lethal *C. allicinum* infection in a frog and highlights the role of this *Cladosporium* sp. in chromoblastomycosis.

## 1. Introduction

*Cladosporium* spp. (Dothideomycetes, Cladosporiaceae), are eso-saprophytic fungi spread worldwide in every kind of terrestrial and marine environment [1,2]. The genus comprises at least 218 species, including common endophytes, plant pathogens, biocontrol agents for plant diseases, and strains causing food spoilage in storage at low temperatures [2,3,4,5]. To the best of our knowledge, reportage of the occurrence of *Cladosporium* spp. infections in human and animals is very limited. Nonetheless, these fungi usually cause allergic rhinitis and even life-threatening exacerbations in asthmatic patients [6,7], eventually leading to opportunistic phaeohyphomycosis (PHM) in humans and animals [2,8,9]. The term PHM is usually linked to infections caused by dematiaceous fungi of different genera (i.e., *Phialophora, Cladosporium, Xylohypha, Curvularia, Exserohilum, Bipolaris, Lecythophora, Alternaria*), which are characterized by the presence of brown pigmented hyphae and yeasts in the histological section of tissues and by the low immunological competence of the host [10,11]. Among PHM, chromoblastomycosis (CBM) is one of the most prevalent invasive fungal infections caused by melanized or brown-pigmented fungi in the form of muriform cells, which causes pyogranulomatous inflammation and fibrosis in host tissues [12]. This infection usually affects immunocompetent rural workers who have a history of local trauma with decaying plant debris. CBM was added to the list of neglected tropical diseases of the World Health Organization in the year 2017 [13].

The etiological agents of CBM belong to a single order in the fungal kingdom, the Chaetothyriales, and include the genera *Fonsecaea, Phialophora*, *Cladophialophora, Exophiala* and *Rhinocladiella.* However, several other genera/species (i.e., *Phialophora, Cladosporium, Xylohypha, Curvularia, Exserohilum, Bipolaris, Lecythophora, Alternaria*) are also rarely and incorrectly reported as aetiological agents of this fungal disease [11,14]. CBM is rarely reported in Europe as an important mycosis [15]. The prevalence for human infection in endemic areas ranged from 1:6800 (Madagascar) to 1:8,625,000 (United States) [12]. However, during the last decades, both CBM and PHM have also increasingly been reported in animals and mainly in cold-blooded vertebrates [16]. Among cold-blooded vertebrates, frogs are more susceptible to dematiaceous fungi especially when they are debilitated and stressed [16,17]. Two forms of infection—cutaneous and disseminated—have been reported in both wild and captive animals [16,18]. Disseminated systemic mycosis was reported as an epizootic among a number of wild and captive frogs (*Hyla caerule*, *Pternohylaf odiens*, *Phyllobatest rinitatis*, *Rhacophorus* spp., and *Hyla septentrionalis*) in the United States, with granulomatous inflammation of the internal organs [16]. Since pigmented hyphae in multiple organs with mild cell necrosis and minimal inflammatory cell response were usually observed in the histological section of the tissues, these infections were classified as PHM. Although CBM and PHM can be differentiated on the basis of the results of histopathological examinations and the immunological competence of the hosts, both diseases in animals have a poor prognosis, most likely due to incorrect or delayed diagnosis and/or treatment [10,11,12,19,20].

Therefore, animal case reports are important for filling knowledge gaps in the diagnosis and treatment of this rare fungal infection. This study describes the first case report of a lethal disseminated *Cladosporium allicinum* infection in a captive bullfrog (*Pyxicephalus adspersus*) in Europe.

## 2. Case Report

**Figure 1:**
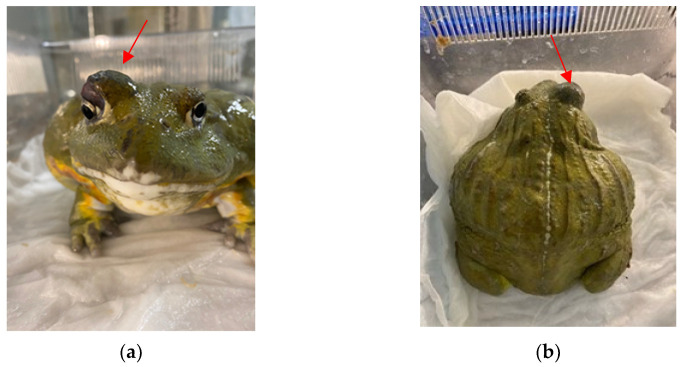
Cutaneous nodule deforming the upper profile of the right orbit: frontal (**a**); and dorsal (**b**) view.

**Figure 2:**
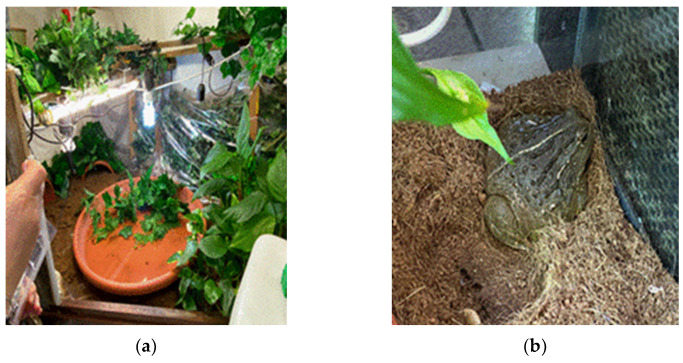
Environment housing adult bullfrogs: Terrarium equipped with a wet area (**a**) and a dry area (**b**) consisting of coconut fibre bedding and environmental enrichment (plants and shelters).

In November 2021, an adult male African bullfrog was referred to a veterinary clinic in Turin with clinical signs of lethargy and the presence of a slow-growing cutaneous nodule deforming the upper profile of the right orbit (Figure 1a,b). The animal was bred in captivity in northern Italy and kept in a terrarium together with ten other adult bullfrogs of both sexes. The amphibians were reared under good conditions of hygiene, lighting (1day/night cycle of 12 h on, 12 h off) and humidity (70%), and were kept in a terrarium with coconut fibre litter and a large wet area with a few centimeters of water. The environment was sprayed with water about every 12 h and kept at a temperature of 22–24 °C (Figure 2a,b). Animals were fed with live bait (mice, cockroaches, and crickets) and the owner reported no history of trauma or injury. Complete X-ray with a dorso-ventral projection and fine needle aspiration from the lesion using a 22-gauge needle were performed. Following cytological examination suggesting a possible fungal infection, surgery was performed, and tissue samples were collected for histological and microbiological investigations. For the surgical procedure, the bullfrog was anaesthetized by injecting the coelomic cavity with propofol (10 mg/kg I.C) and maintained with isoflurane (2% in mask) in association with locally injected lidocaine. Post-operative treatments included enrofloxacin (Baytril 22.7 mg/mL, Bayer, Shawnee Mission, KS, USA) at 10 mg/kg administered intra-coelomically, and meloxicam (0.2 mg/kg) for analgesia in the first 24 h. After surgical excision, collected tissue samples (i.e., approximately 1 mm × 1 mm × 1 mm) were fixed in 10% phosphate-buffered formalin for histopathological examination and stored in e-Swab^®^ (Copan, Italy) for microbiological examination. Environmental and skin swab samples from some frogs in the same terrarium were collected. Specifically, terrarium atmospheric air samples were collected by opening sterile plate containing specific media (i.e., Sabouraud Dextrose agar with 0.5% chloramphenicol –SAB; Becton Dickinson GmbH, Germany) in the terrarium for 15 min, while e-Swab^®^ (Copan, Italy) were used for surfaces and skin of animals. All the collected samples were sent for microbiological examination. After laboratory suspicion of fungal infection following both microbiology and histopathology evaluations, climbazole therapy by transcutaneous absorption BID was performed. During therapy, the frog showed no improvement, maintaining a lethargic attitude, anorexic and showing no substantial weight loss. After 30 days from the start of medical treatment, the animal died spontaneously, and a full necropsy was performed. Tissue sections (approximately 4 µm thick) from major organs (i.e., lungs, kidney, intestines, heart, and brain) were obtained using routine histological techniques and used for further histopathological and microbiological investigations.

## 3. Materials and Methods

For cytological examination, the smeared specimens were air dried and stained with Romanowsky stain (May-Grunwald-Giemsa, Merk KGaA, Darmstadt, Germany). For histopathological examination, the samples were collected and immersed in buffered 10% formaldehyde solution for fixation. After fixation, the tissues went through dehydration in graded alcohol (70–100%), cleared in xylene, embedded in paraffin wax and microtome sectioned at 4 μm were stained with Haematoxylin and eosin (H&E) [21].

Needle aspirate samples, tissue samples, and environmental samples were cultured onto SAB, Trypticase Soy Agar II with 5% Sheep Blood and MacConkey (Becton Dickinson GmbH, Germany) and incubated at 25 °C, 30 and 37 °C for seven days. No bacterial growth was observed while the fungal culture was positive. Colonies grown on SAB were subcultured onto Potato Dextrose Agar (PDA; Liofilchem, Italy) and identified to genus level based on macroscopic and microscopic examination [22]. The identification was confirmed by mass spectrometer MALDI-TOF Biotyper^®^ (Bruker Daltonics GmbH & Co. KG., Bremen, Germany) and by molecular methods. Molecular biology investigations were performed concurrently from the paraffin-embedded tissue sample and from the isolated fungal colony. Specifically, genomic DNA was isolated from samples using the DNeasy Blood & Tissue Kit (QIAGEN, Hilden, Germany) following manufacturer instructions for fixed tissues and for fungi.

The primers ITS1 (5′-TCCGTAGGTGAACCTGCGG-3′) and ITS4 (5′-TCCT CCGCTTATTGATATGC-3′), and EF1-1018F (5′-GAYTTCATCAAGAACATGAT-3′) and EF1-1620R (5′-GACGTTGAADCCRACRTTGTC-3′) were used to amplify the ITS1-5.8S-ITS2 region and the translation elongation factor 1-α (TEF1α) gene, respectively [23]. Two separate PCR amplifications were carried out in 50 μL using the DreamTaq PCR master mix (Thermo Scientific, Monza, Italy), 100 ng of genomic DNA template, and 0.5 μM of each primer described above.

The PCR cycling parameters used for ITS amplification consisted of an initial heating to 95 °C for 5 min, 35 cycles of denaturation at 94 °C for 1 min, annealing at 55 °C for 40 s, and extension at 72 °C for 45 s, followed by a final extension step of 10 min at 72 °C. PCR conditions for amplifying partial TEF1α gene using primers EF1-1018F and EF1-1620R differed only by their annealing temperature (60 °C instead of 55 °C) and increased cycle extension time (60 s instead of 45 s per cycle).

PCR products were enzymatically purified using the ExoSAPT-IT reagent (Thermo Fisher Scientific, Italy) and bidirectionally sequenced at the Eurofins Genomics (Ebersberg, Germany) using standard Sanger sequencing and the same primer set used for PCR. Raw nucleotide sequences were edited using the FinchTV v1.4 software (GeoSpiza Inc., Seattle, WA, USA). For each genetic marker, final DNA consensus sequence was generated by matching both forward and reverse sequencing traces and then extracted in a text-based FASTA format, which was used for the taxonomic recognition of the fungal isolate by BLASTn search [24] of similar sequences deposited in the GenBank [25] and the MYCOBANK [26] databases.

## 4. Results

**Figure 3:**
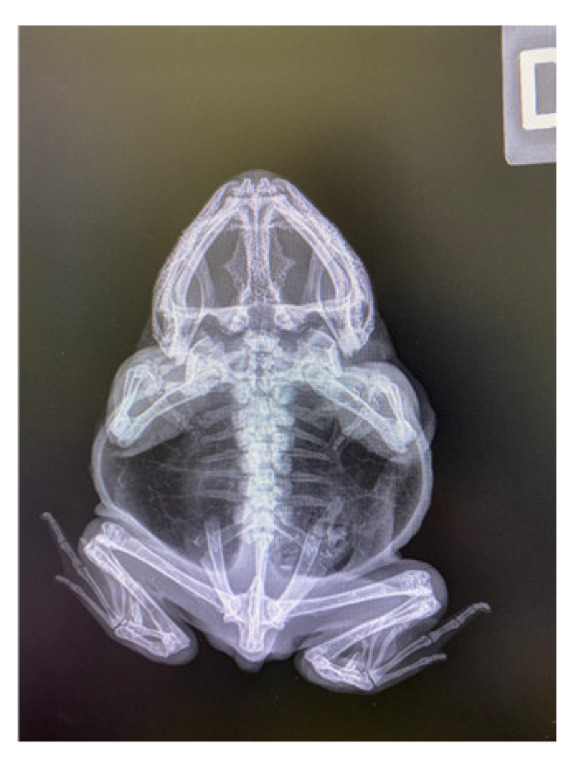
X-Ray, ventral dorsal projection of the frog, absence of bony changes in the right orbit.

X-Ray revealed that the mass was limited to soft tissues without bone involvement (Figure 3).

**Figure 4:**
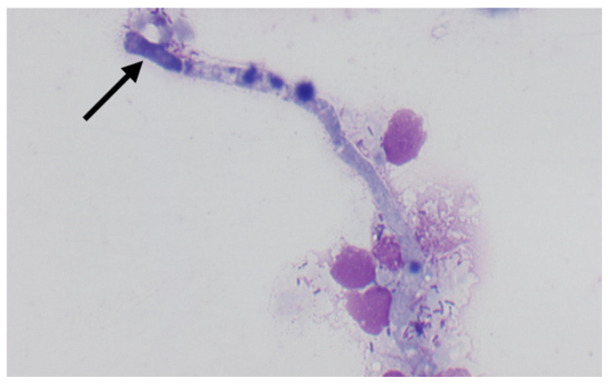
Cytological examination: Neutrophilic inflammation with macrophages with the presence of pigmented septate fungal hyphae (arrowed) ×100.

**Figure 5:**
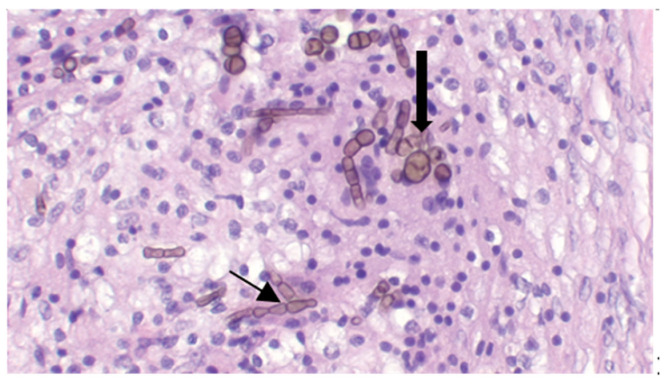
Histopathological examination: diffuse granulomatous inflammation, pigmented fungal hyphae (thin arrows) and muriform bodies (thick arrows) ×40 H&E.

On cytological examination, specimens were poorly cellular differentiated and showed neutrophilic-macrophages inflammation with the presence of septate fungal hyphae (Figure 4). At histopathological examination, pigmented hyphae, and structures consistent with muriform bodies were found on a background of diffuse granulomatous inflammation from collected tissue samples (Figure 5).

**Figure 6:**
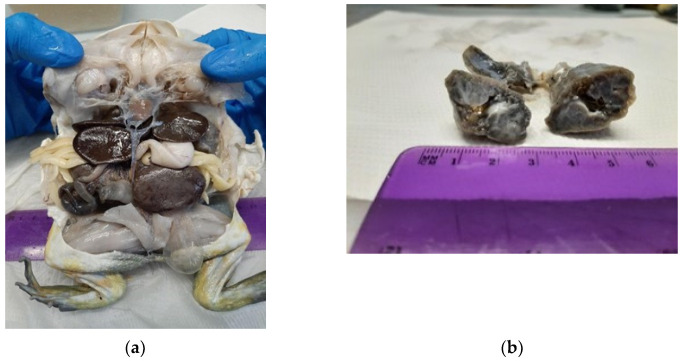
Nodular and diffuse granulomatous lesions in liver, kidneys, lungs and large intestine (**a**); and right lung, irregularly thickened, with grey-brownish lesions (**b**).

At necropsy, a large mass of granulating grey-brownish tissue altered the normal architecture of the inner parts of the head. The mass protruded within the right portion of the oral cavity and extended through the hard palate to invade the cranial cavity effacing the nervous tissue, finally reaching, and obliterating the right orbit. Similar tissue formed two nodules in the liver, expanded and effaced the kidneys, and irregularly thickened the lungs with the right lungs more severely affected (Figure 6a,b). Histopathological examination of tissues collected during necropsy revealed the presence of a granulomatous mass with hyphae and muriform bodies effacing the architecture of the head tissues. Similar multifocal nodular to coalescing and diffuse lesions were found in the liver, kidneys, lungs, and large intestine.

**Figure 7:**
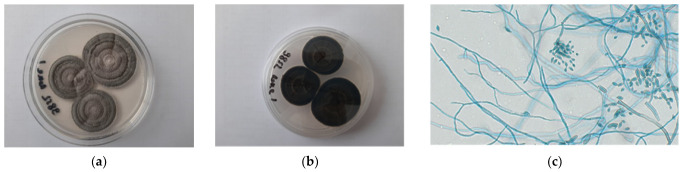
*C. allicinum* culture, macroscopic olive black filamentous colonies, right (**a**); and reverse (**b**), after 7 days of incubation at 25 °C on Sabouraud Dextrose and microscopic observation in lactophenol: septate hyphae and numerous and branched acropetal chains conidia ×40 (**c**).

No fungal and bacterial growth was observed from samples cultured in the media incubated at 37 °C. Only olive black filamentous colonies were isolated in pure culture from all the sampled tissues, after incubation at 25 °C and 30 °C. The microscopic examination revealed brown septate hyphae and numerous similarly pigmented conidia. Conidia were arranged in branched acropetal chains, being smooth, verrucose or echinulate presenting a distinct dark hilum. On the basis of the macroscopic and microscopic examination, the isolate was identified as *Cladosporium* spp. (Figure 7a–c). Using MALDI-TOF procedure, the fungal isolate was identified as *Cladosporium herbarum* with a match scoring of 1.72 (low confidence identification). The results of molecular analysis performed in parallel from the paraffin-embedded tissue sample and from the isolated fungal colonies showed same results and all the strains were identified as *C. allicinum*. However, a proper species identification was possible only by partial sequencing of the TEF1α gene as the high level of sequence similarity observed by comparing the ITS region with several sequences deposited in the Genbank, and/or MYCOBANK database, did not provide any indication to resolve the genetic relationships among our and many other *Cladosporium* species. Indeed, the BLASTn search of the Genbank database using our ITS sequence as query, showed 100% nucleotide identity with many different *Cladosporium* spp. Similar results were also obtained by searching the MYCOBANK database. On the other hand, BLASTn analysis of the TEF1α sequence revealed that the greatest similarity (≥99.77%; e-value: 0) was shared with only three Genbank (MN054844, DQ677891, DQ677918) and one MYCOBANK (DQ677918) TEF1α sequences, one of which (DQ677918) belonged to the reference strain *C. allicinum* CBS 399.80 [1,2]. The other two Genbank sequences (MN054844 and DQ677891), although currently associated with fungal isolates identified as *Cladosporium* sp., probably represent additional isolates of *C. allicinum*. The sequences were deposited in the GenBank database with accession numbers OQ03117 for TEF1α and OP965390 for ITS.

## 5. Discussion

To the best of our knowledge, this is the first reported Italian case of lethal *Cladosporium* spp. infection in a frog, pointing out its role in CBM.

*Cladosporium* species are ubiquitous, found everywhere in soil, on decaying vegetation and rotten wood but the disease is primarily prevalent in the hot and humid climates of tropical and subtropical regions [27]. The organism grows optimally within a temperature range of 18–30 °C; however, the growth is not possible at temperature above 35–37 °C for *Cladosporium* spp. [28,29].

Both in humans and animals, the disease is characterized by: (I) traumatic inoculation of fungus from an environmental source leading to an initial cutaneous lesion at the inoculation site; (II) chronic and progressive cutaneous and subcutaneous tissue involvement associated with granulomatous reactions; and (III) haematogenous dissemination (especially with *Cladosporium trichoides* infections) to distant sites including brain to produce so called “brain abscess syndrome” [12]. Usually, the disease is associated with a non-protective T helper type 2 (Th2) immune response with ineffective humoral immunity in humans and mammals [12]. In cold-blooded animals, disease occurs predominantly in stressed animals, mainly after removal from their natural habitat [30]. Additionally, the presence of aromatic toxins in amphibian skin is considered a predisposing factor for skin diseases [12,16]. In this study we describe a *C. allicinum* infection in an African bullfrog (*Pyxicephalus adspersus*) reared in captivity. These animals are large, classic-looking frogs that are native to Africa but found in homes as pets around the world. Nevertheless, their captive maintenance is usually challenging, and many frogs die soon after acquisition usually due to stress-related illness or trauma [31,32]. The difficulty in the breeding management of these animals might have favored the fungal infection in the skin of the animal herein investigated. Nevertheless, the finding of negative results in the fungal cultures of terrarium might suggest that the spread of *C. allicinum* was mediated by the air, live baits, or the breeder’s hand. The lower body temperature and the skin features of this animal species might have favored the systemic spread of *C. allicinum* to other tissues.

The finding of systemic spreading of the fungus differs from the typical localizations of *Cladosporium* spp. infection in humans and mammals which was usually localized in the skin [33], whereas brain involvement has been reported only sporadically in humans, dogs, and sheep [34,35].

As for diagnostic procedures, this study confirmed the usefulness of cytology and histopathology with routine staining to diagnose infection by dematiaceous fungi. The presence of muriform cells observed at the histopathological examination of tissues of this animal, suggests a role of *C. allicinum* in CBM. Indeed, the histopathological picture observed in this study was never observed in any *Cladosporium* spp. infections, being mainly associated with fungi belonging to *Fonsecaea*, *Cladophialophora* and *Phialophora* [35]. CMB and PHM are less common fungal infections caused by dark-pigmented fungi but the virulence factors, such as muriform cells, play an important role in the pathogenesis of CMB, thus causing infections in hosts with fully functioning immunity [35]. In addition, the current study confirms the considerable difficulty in identifying *Cladosporium* spp. [1,36,37,38,39,40]. In this study, the use of ITS sequencing failed to correctly identify the strain at species level confirming previous data [2] which reported that this locus has limited resolution below genus level in *Cladosporium*, and it is unable to distinguish many species, especially in the *C. cladosporioides* and *C. herbarum* species complexes [2]. However, sequence analysis of the TEF1α gene provided an accurate identification, with higher resolution to ITS, corroborating its use as an additional universal DNA barcode marker for fungi [23].

As for therapy, in this case, climbazole by transcutaneous absorption failed to resolve the pathology, probably due to the delay in the initiation of treatment. Nevertheless, this finding confirms the difficulty in the management of these mycoses. Despite the availability of various systemic antifungal drugs in human medicine, no guidelines of antifungal drugs are available for many animal species, including frogs. In addition, there is a paucity of information regarding antifungal susceptibility profile data for *Cladosporium* spp. [9]. Case reports have shown a favorable outcome using azole-based therapies, and itraconazole and posaconazole seem to be the most active drug [12,16].

Nevertheless, the employment of antifungal drugs with physicochemical methods (e.g., surgery, thermotherapy, laser therapy, and photodynamic therapy) or the combination of both to treat the disease in humans is still related to a low cure rate and a high relapse rate, especially in chronic and extensive infections [41]. For animals, especially frogs, management practices such as handling, crowding, transporting, fluctuating temperatures, poor water and stable quality and other husbandry deficiencies should be accurately controlled in order to prevent infection and to reduce the risk of transmission to other animals or humans.

## 6. Conclusions

This is the first case report of a fatal *C. allicinum* infection in a frog showing its role in CBM. The treatment herein proposed was not successful most likely due to delay in the diagnosis and drug administration. In the management of this extensive disease, desirable goals are early diagnosis, species-specific therapy, and adequate management strategies to prevent infection and reduce the risk of transmission.

## Data Availability

The data presented in this study are available on request from the corresponding author.
